# Parkinsonism in idiopathic normal pressure hydrocephalus: is it time for defining a clinical tetrad?

**DOI:** 10.1007/s10072-022-06119-3

**Published:** 2022-06-01

**Authors:** Giovanni Mostile, Alfonso Fasano, Mario Zappia

**Affiliations:** 1 Department “G.F. Ingrassia”, Section of Neurosciences, University of Catania, Via Santa Sofia 78, 95123 Catania, Italy; 2 Oasi Research Institute-IRCCS, Troina, Italy; 3 Edmond J. Safra Program in Parkinson’s Disease, Morton and Gloria Shulman, Movement Disorders Clinic, Toronto Western Hospital, UHN, Toronto, ON Canada; 4 Division of Neurology, University of Toronto, Toronto, ON Canada; 5 Krembil Brain Institute, Toronto, ON Canada; 6 CenteR for Advancing Neurotechnological Innovation to Application (CRANIA), ON Toronto, Canada

**Keywords:** Idiopathic normal pressure hydrocephalus, Gait disorders, Parkinsonism

## Abstract

**Background:**

Association between parkinsonism and idiopathic normal pressure hydrocephalus (iNPH) still remains debated. There is already plenty of evidences in the literature suggesting that this clinical sign can be considered as an integral part of the clinical spectrum of iNPH patients.

**Methods:**

We reviewed the possible pitfalls in the core clinical definition of iNPH based on available international diagnostic criteria, phenomenology of parkinsonism in iNPH, and neuroimaging supporting the presence of parkinsonism in iNPH.

**Conclusions:**

We argue that the diagnostic definition of the iNPH “triad” should be possibly reconsidered as a “tetrad” also including parkinsonism.

## Pitfalls in the core clinical definition of iNPH

The definition of normal pressure hydrocephalus (NPH) comes from early anecdotal descriptions by Salomon Hakim and Raymond Adams [1]. Their three reported cases already appeared heterogeneous in terms of etiology, anamnestic data, and clinical presentation. Yet, they coined the famous clinical triad of impaired walking and balance, urinary incontinence, and cognitive decline. Later on, the term “idiopathic” has been adopted to define those conditions with ventricular enlargement and normal cerebrospinal fluid (CSF) pressure in the absence of identifiable acquired causes of NPH.

To date, at least four diagnostic guidelines based on clinical, radiological, and instrumental features have been published [2–5]. Despite this, diagnostic problems still persist. The presence of further clinical signs in addition to the aforementioned triad calls into question the accuracy of the proposed criteria when used in the differential diagnosis with other neurodegenerative conditions, primarily Parkinson’s disease (PD) but also other atypical parkinsonisms.

The American-European guidelines proposed in 2005 include two levels of diagnostic accuracy [2]. The diagnosis of “possible” idiopathic NPH (iNPH) can be made by the presence of at least one sign of the classical triad in the context of ventriculomegaly on brain imaging. On the other hand, gait and balance problems are mandatory for the diagnosis of “probable” iNPH, when associated with at least one other area of impairment in cognition, urinary symptoms, or both, specific supportive neuroimaging features as well as a CSF opening pressure documented in the 5–18 mmHg (or 70–245 mmH_2_O) range. Although several characteristics of gait impairment required for diagnosis are specified, it is also stated that they “may coexist with other conditions” documented in the same patient (including neurodegenerative conditions such as PD), but “should not be entirely attributable” to them. Furthermore, although not included as a diagnostic criterion, “parkinsonism” is considered a possible detectable clinical feature in iNPH, with a variable response reported to L-dopa treatment and shunt procedures. No specific information is provided on the differential diagnosis with PD.

The third edition of the Japanese guidelines supports a diagnosis of “possible” iNPH based on at least two clinical signs of the classical triad [5]. The level of diagnostic accuracy increases to “probable” when all of the following requirements are met: a normal CSF opening pressure (≤ 200 mmH_2_O), a response from 24 h to 1 week after CSF tap test or external lumbar drainage and the presence of neuroradiological patterns such as “DESH” (i.e., “disproportionately enlarged subarachnoid-space hydrocephalus”). In probable iNPH, gait impairment is characterized by small shuffling steps and dynamic instability on walking and turning. A diagnosis of “definite” iNPH requires a clinical response to shunting procedures. The clinical diagnosis is substantially entrusted to the presence of a gait disturbance and the frequent lack of a complete clinical triad is highlighted. However, even these guidelines fail to characterize the specific gait features useful in the differential diagnosis with degenerative parkinsonisms.

## Evidences of parkinsonism in iNPH

Parkinsonism is an often overlooked clinical feature of iNPH. From the earliest descriptions, it seemed clear that the presence of parkinsonism could not preclude a clinical response to shunt procedures, nor that the gait abnormalities could be different from those observed in PD [6]. Akinesia in iNPH has been reported in nearly 70% of cases, with bradykinesia and postural instability being the main observed features [7, 8]. Hypokinetic motor deficit may involve the upper limbs in iNPH, sharing the same characteristics observed in PD [9]. Several types of parkinsonism, including a symmetrical lower body parkinsonism, but also an asymmetrical and even dominant upper body phenotype, may be part of the clinical presentation of iNPH [10].

Although the clinical response to L-dopa was generally reported as poor in these patients, a motor response was documented by acute challenge testing as well as after 4–6 weeks of dopaminergic treatment with L-dopa up to 1250 mg/day, before testing patients with iNPH to shunt response [11]. The response of parkinsonian signs to tap test or ventriculo-peritoneal shunt has been poorly explored in iNPH associated with parkinsonism and the results have been inconsistent, even because parkinsonism has only rarely been considered an outcome measure [12]. Unified Parkinson’s Disease Rating Scale (UPDRS) motor score reduction has been reported to vary between 12 and 18% after tap test or external lumbar drainage [12, 13]. Following ventriculo-peritoneal shunt procedures, a response rate of 37% after 1 week and 25% after 1 year has been estimated [14, 15], with over 60% of patients showing greater than 30% improvement on UPDRS motor score [15].

There are uncertainties in the definition of “parkinsonism” and “gait disturbance” in iNPH. The definition of “parkinsonism” is provided by the Movement Disorder Society (MDS) diagnostic criteria for PD as prerequisite for the clinical diagnosis [16]. Parkinsonism should be distinguished from clinical PD since it can be detected in other neurodegenerative conditions such as tauopathies [16]. Parkinsonism is based on the presence of bradykinesia as a cardinal feature, in combination with resting tremor, rigidity, or both. “Bradykinesia” has also been strictly defined as slowness of movement and a decrease in amplitude or speed as movements continue (the so-called “sequence effect”), being better characterized for PD and less for other atypical parkinsonisms [16, 17]. Although routinely evaluated and documented as bradykinesia of the limbs, it can also be described during gait, with a progressive reduction of the step length leading to motor blocks (“freezing” phenomenon) [17, 18].

Human locomotion is the result of the modulation of the central spinal pattern generators induced by the supraspinal regions, which include the pontomedullary reticular formation, the mesencephalic locomotor region, the basal ganglia and the frontal cortical regions [19]. When the basal ganglia and supplementary motor area (SMA) loops are disrupted, self-initiated movements are affected leading to freezing of gait and the dependence of walking on external cues to maintain locomotor thrust [19]. In this scenario, it could be argued that pathological conditions affecting both the frontal lobe and the basal ganglia may share the same gait dysfunctions [19, 20]. Clinically, the presence of lower body parkinsonism with postural instability can in fact lead to a diagnostic overlap between iNPH and PD, vascular parkinsonism and atypical parkinsonism such as progressive supranuclear palsy (PSP) [21, 22]. While some studies have proposed characteristic gait features that discriminate iNPH from PD, such as enlarged stride width, larger foot angles, and lack of improvement with cues [23], others have reported similar patterns in both conditions [24]. A more recent gait analysis study paper found that gait in iNPH and PSP largely overlaps unless specific dual task conditions are considered [25]. Furthermore, there is some evidence that iNPH patients with and without parkinsonian symptoms may report similar improvement after the diagnostic CSF tap test [26]. On the other hand, vascular parkinsonism, a still debated nosological entity, has been reported to respond to CSF drainage when associated with ventriculomegaly [20, 27, 28].

## Neuroimaging support of parkinsonism in iNPH

Parkinsonism in iNPH has been correlated with structural data from neuroimaging, including binding of the striatal dopamine reuptake transporter [29], brain ventricular enlargement estimated from morphometric measurements [14], and injury burden white matter, which may even improve after shunt procedures [30].

It has mainly been postulated that parkinsonian features in iNPH could be related to several mechanisms responsible for the disconnection between cortico-subcortical networks at different levels: midbrain, pallido-thalamic fibers, or fibers that connect the thalamus to the SMA [31]. Evidence supporting possible pathophysiological mechanisms inducing parkinsonism in iNPH has been provided by functional neuroimaging.

A functional magnetic resonance imaging (fMRI) study demonstrated a significant enhanced SMA activity during finger motor performance in iNPH patients after CSF drainage, hypothesizing that improved motor performance after CSF subtraction could be related to a direct effect on cortical areas involved in the preparation and monitoring of movement [32]. SMA and its connections with the basal ganglia have been also examined in patients with PD, with evidence of their hypoactivity on fMRI during the pharmacological “off” phase, being restored after taking L-dopa [33], thus indicating similar pathogenetic bases.

Furthermore, a postsynaptic dysfunction of D2 receptors in the dorsal putamen and nucleus accumbens has been demonstrated in a positron emission tomography (PET) study including patients with iNPH and parkinsonism [34], which is restored after shunt [35]. The results suggest that down-regulation of the postsynaptic D2 receptor could be related to a dysfunction of the cortico-striatal network due to hydrocephalus. This hypothesis on the mechanical effect exerted on the striatum by ventriculomegaly, which should be prominent in the caudate nucleus leading to the downregulation of dopaminergic transporters, has also been supported by single-photon emission computerized tomography (SPECT) studies [29, 36, 37]. In a recent paper striatal dopamine reuptake transporter density has been shown to differentiate iNPH patients with prevalent imbalance from those with major locomotor impairment, which also demonstrated a significant association between parkinsonism and striatal uptake only for the latter phenotype [38]. Gait and caudate radiotracer binding improved in both phenotypes after surgery, while parkinsonism and putamen radiotracer density improved in shunted patients with major locomotor impairment. Study patients presented no response to L-dopa on parkinsonian features [38]. This finding further supports the close link between gait disturbance and parkinsonism in iNPH. Evidence of Lewy body pathology in iNPH was also supported by 123I-metaiodobenzylguanidine myocardial scintigraphy studies, which demonstrated cardiac sympathetic abnormality in some patients [39].

## Proposals for a redefinition of iNPH diagnostic criteria

Based on the reported evidence, which includes: (a) the underestimated real prevalence of parkinsonism in iNPH; (b) the difficulty in identifying iNPH-specific gait parameters without taking into account the possible features seen in “lower body parkinsonism”; (c) the response of parkinsonian signs to surgical shunting procedures; (d) the rare but possible response of parkinsonian signs in iNPH to dopaminergic treatment; (e) the neuroimaging evidences supporting possible pathophysiological mechanisms inducing parkinsonism in iNPH, it is necessary to re-evaluate the classic clinical diagnostic triad for iNPH, including the presence of parkinsonism as a component of a tetrad of symptoms beside to gait disturbances, cognitive impairment, and urinary dysfunction. Definition of specific clinical aspects and instrumental supporting features of parkinsonism associated to iNPH should be discussed among study groups and experts in this field.
